# Langasite as Piezoelectric Substrate for Sensors in Harsh Environments: Investigation of Surface Degradation under High-Temperature Air Atmosphere

**DOI:** 10.3390/s21175978

**Published:** 2021-09-06

**Authors:** Thierry Aubert, Ninel Kokanyan, Omar Elmazria

**Affiliations:** 1 Université de Lorraine—CentraleSupélec, LMOPS, F57070 Metz, France; thierry.aubert@univ-lorraine.fr (T.A.); ninel.kokanyan@centralesupelec.fr (N.K.); 2 Université de Lorraine—CNRS, Institut Jean Lamour (UMR 7198), F54000 Nancy, France

**Keywords:** langasite, temperature sensors, harsh environments

## Abstract

Langasite crystals (LGS) are known for their exceptional piezoelectric properties at high temperatures up to 1000 °C and more. In this respect, many studies have been conducted in order to achieve surface acoustic wave (SAW) sensors based on LGS crystals dedicated to high-temperature operations. Operating temperatures of more than 1000 °C and 600 °C for wired and wireless sensors, respectively, have been reached. These outstanding performances have been obtained under an air atmosphere since LGS crystals are not stable in high-temperature conditions under a low-oxygen atmosphere due to their oxide nature. However, if the stability of bulk LGS crystals under a high-temperature air atmosphere is well established, the surface deterioration under such conditions has been hardly investigated, as most of the papers dedicated to LGS-based SAW sensors are essentially focused on the development of thin film electrodes that are able to withstand very elevated temperatures to be combined with LGS crystals. Yet, any surface modification of the substrate can dramatically change the performance of SAW sensors. Consequently, the aim of this paper is to study the stability of the LGS surface under a high-temperature air environment. To do so, LGS substrates have been annealed in an air atmosphere at temperatures between 800 and 1200 °C and for durations between one week and one month. The morphology, microstructure, and chemical composition of the LGS surface was examined before and after annealing treatments by numerous and complementary methods, while the surface acoustic properties have been probed by SAW measurements. These investigations reveal that depending on both the temperature and the annealing duration, many defects with a corolla-like shape appear at the surface of LGS crystals in high-temperature prolonged exposure in an air atmosphere. These defects are related to the formation of a new phase, likely an oxiapatite ternary compound, the chemical formula of which is La_14_Ga_x_Si_9−x_O_39−x/2_. These defects are located on the surface and penetrate into the depth of the sample by no more than 1–2 microns. However, SAW measurements show that the surface acoustic properties are modified by the high-temperature exposure at a larger deepness of at least several tens of microns. These perturbations of the LGS surface acoustic properties could induce, in the case of LGS-based SAW sensors operating in the 434 MHz ISM band, temperature measurement errors around 10 °C.

## 1. Introduction

### 1.1. Conventional Piezoelectric Crystals for High-Temperature SAW Applications

Surface acoustic wave devices (SAW) have been key elements of telecommunication systems, including mobile phones, for more than three decades. One of the challenges that is currently being faced with these kinds of applications is to minimize, by design or materials considerations, the influence of environmental parameters on device performance. Indeed, SAW devices can be very sensitive to many parameters, including the temperature. This drawback has been rapidly seen as an opportunity by the SAW community, as it means that SAW devices can be used as sensors. In this respect, SAW devices have great added value, as they are passive components. They thus can be wirelessly interrogated without any embedded electronics or power source. They just reflect a small fraction of the incident signal coming from the interrogation system. Such sensors are very useful in situations where wires are to be avoided and conventional wireless sensors cannot be used. SAW sensors are thus particularly indicated to remotely monitor the physical parameters of moving parts placed in high-temperature (HT) environments, typically above 250 °C. Many industrial sectors, including metallurgy, glass, aeronautics, automotive, space, nuclear, or oil extraction, are particularly concerned by this technology. This idea to develop high-temperature SAW sensors appeared in the middle of the nineties. One of the first reference papers on the topic was written by Hornsteiner et al. in 1998 [1]. In this paper, the authors list the potential piezoelectric substrates that are able to operate in HT conditions. Quartz, lithium tantalate, or lithium niobate, which are the three main substrates used for telecommunication applications, are all limited for HT applications. To date, the most advanced SAW sensors based on these well-known crystals are made from lithium niobate and can be used up to approximately 400 °C [2]. At higher temperatures, a segregation process occurs in lithium niobate crystals, leading to the apparition of lithium triniobate LiNb_3_O_8_ at the substrate surface [3]. Recent work shows that LiNbO_3_ could be considered to operate up to 600 °C but for limited durations [4], and the use of stoichiometric LiNbO_3_ (s-LN) substrate makes it possible to exceed this limit for even longer durations [5]. Nevertheless, s-LN substrates remain expensive, and the cuts of interest for SAW applications are not commercially available.

### 1.2. Langasite: A Suitable Piezoelectric Material for HT SAW Applications

The other main interest of Ref. [1] was to experimentally explore the potential for HT applications of a relatively new piezoelectric material at the time, namely langasite La_3_Ga_5_SiO_14_ (LGS). The synthesis of LGS crystals by the Czochrlaski method was first achieved in 1981 in the USSR [6]. The process was then continually improved so that good quality wafers up to 4 inches in diameter were available in the nineties. These efforts were motivated by the intermediate electromechanical coupling factor K^2^ of LGS, in the range of 0.4–0.5%, which allowed the realization of medium bandpass SAW filters, in complement of narrow bandpass filters based on quartz, and large bandpass filters based on lithium niobate. However, LGS crystals also have a great potential for HT applications, as they do not undergo any phase transition up until its melting temperature, located at 1473 °C. Thus, LGS keeps its piezoelectric properties up to very high temperatures, as demonstrated by Fritze, who generated piezoelectrically excited bulk acoustic waves (BAW) in LGS up to 1400 °C [7]. Regarding SAW applications, in Ref. [1], Hornsteiner et al. managed to measure a SAW signal up to 1085 °C, which suggested, for the first time, the possibility of developing SAW sensors able to operate at temperatures of 1000 °C or even more. Consequently, LGS was then intensively studied for such purpose. Several remarkable results were achieved. In particular, a SAW signal was measured on a LGS based SAW device up to 1140 °C [8], whereas Pereira Da Cunha et al. measured a stable SAW signal at 800 °C for more than five and a half months [9], thus confirming the great potential of LGS for HT SAW applications. Several studies also provided evidence of some limitations of LGS crystals. The first one is that SAW propagation losses in LGS increase very quickly together with the temperature and the frequency, prohibiting its use in GHz-range ISM bands [10]. Consequently, the most advanced achievement regarding wireless HT LGS-based SAW sensors was realized with SAW resonators operating in the 434 MHz ISM band able to work up to 700 °C for tens of hours [11]. This result constitutes the state of the art regarding wireless SAW sensors operating in ISM bands. Many issues regarding electrodes, packaging, interconnections, antennas, etc. are still to be fixed so that higher temperature measurements can be reached [12,13]. Moreover, as for any semiconductor crystal, the electrical resistivity of LGS decreases with the temperature, which unfavorably impacts the quality factor of SAW resonators [14]. Eventually, as for many oxides, LGS crystals undergo surface degradations in HT environments with low levels of oxygen. Gallium and oxygen losses have been noticed from 650 °C in such conditions [15]. Moreover, because LGS maintains its piezoelectric properties at very high temperatures and because good-quality wafers are commercially available, LGS is currently one of the very few piezoelectric materials that allow the development of SAW sensors for applications above 600 °C, in particular under an air atmosphere. Thus, research work on LGS as a potential substrate for hash environment applications continues to mobilize scientists, as evidenced by the many recent published papers [12,13,16,17,18,19,20]. In fact, most of the studies dedicated to LGS based SAW sensors are essentially focused on either the development of thin film electrodes able to withstand very elevated temperatures to be combined with LGS crystals [8,9,12,13], the frequency–temperature behavior of various cuts [16,19], or the optimization of the sensor design in order to compensate for the decrease of the quality factor at high temperatures [17]. Finally, apart from this main application as a HT piezoelectric substrate, LGS material is foreseen to play other roles in the SAW domain. Thus, doped LGS thin films are even studied as electrodes for HT SAW applications [20]. Moreover, the trend to densify the integration of RF components and in particular in communication systems, coupled with the increase in operating frequencies to allow for wide bandwidths, imply an extreme miniaturization of SAW devices and therefore a very localized concentration of power. This generates an intense heating of the substrate in the vicinity of the electrodes and thus requires the use of suitable materials [21].

### 1.3. Stability of Langasite Surface under HT Air Atmosphere

This rapid overview of the papers dedicated to the study of LGS for SAW applications shows that the specific study of the surface deterioration of LGS crystals under a HT air atmosphere has hardly been investigated, whereas this phenomenon is quite well documented when it occurs under atmospheres with low levels of oxygen [8,15]. Yet, any substrate surface modification can dramatically change the performance of SAW sensors. One of the only papers on the topic was published by Schulz et al. in 2004. The authors calculated that the surface of LGS crystals placed at 750 °C for 100 days in air can lose 7 × 10^15^ Ga atoms per cm^2^, which represents the quantity of Ga atoms in the first 4 nanometers of the crystal [22]. It can be concluded from this result that the surface of LGS is stable enough that these crystals can be used for SAW applications at 750 °C for several months in an air atmosphere, as the penetration depth of the acoustic wave is several microns. This is in very good correlation with the empiric result obtained by Pereira Da Cunha et al., who operated a LGS-based SAW device for more than 5 months at 800 °C [9]. Thus, LGS crystals could potentially be used at higher temperatures in an air atmosphere, potentially up to 1000 °C or even more. This requires having better knowledge of the surface behavior of LGS crystals at temperatures above 800 °C in an air atmosphere. This is precisely the aim of the present paper.

## 2. Experimental

The study was conducted on Y-cut LGS substrates purchased at Witcore company (Jinan, China). Most of the samples were annealed in an air atmosphere without control of the humidity for one week at temperatures between 800 and 1200 °C in a tube furnace (Carbolite STF 15/180). Some samples were annealed at 1000 °C for a longer time (one month). In order to study the impact of a high-temperature air environment on the surface of LGS crystals, the samples were characterized before and after the abovementioned annealing treatments by different methods. Changes in the surface chemical composition and surface morphology were studied by energy dispersive X-ray spectroscopy (EDXS) and scanning electron microscopy, respectively (SEM, Tescan Vega 2). The surface topology was inspected by atomic force microscopy (AFM, Veeco CP Research). The microstructure of the samples was examined by X-ray diffraction (XRD) in Bragg–Brentano geometry (Brucker D8 Advance—${\mathrm{Cu}}_{{K\alpha }_{1}}$: λ = 1.54056 Å) and Raman spectroscopy (Horiba HR Evolution). The wavelength of the excitation laser was 532 nm, thus leading to a penetration depth of about 1 µm. As Raman spectroscopy enables micronic local measurements, 3D microstructure cartographies were achieved in order to obtain access to the dimensions of some of the observed defects. Finally, SAW measurements were performed at room temperature on delay lines built on reference (non-annealed) substrates and those annealed for one month at 1000 °C. Interdigital transducers (IDTs) were made by conventional photolithography and chemical etching from 100 nm thick aluminium films deposited by the sputtering method. The devices were composed of two identical IDTs, with 50 pairs of fingers and a wavelength λ of 28 μm. The IDT center spacing, acoustic aperture, and finger width to space ratio were 80λ, 40λ, and 1:2, respectively. The SAW propagation path was along the X-direction of the LGS.

## 3. Results and Discussion

### 3.1. One-Week Annealing Process at Temperatures up to 1100 °C

**Figure 1:**
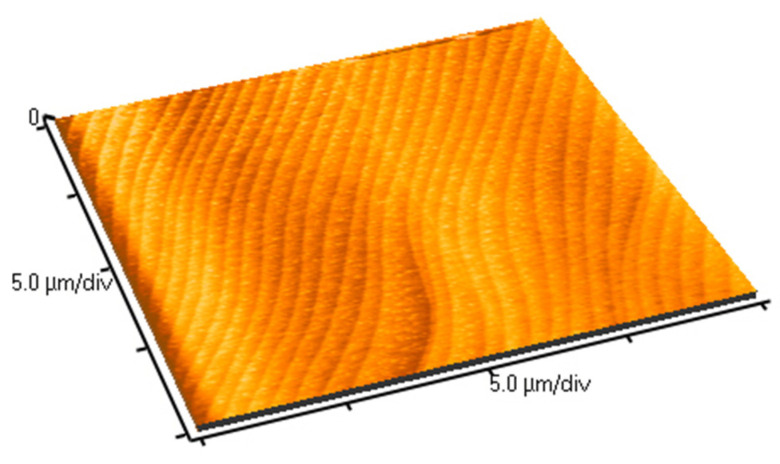
AFM image of LGS crystal surface after one week at 1100 °C under an air atmosphere.

**Figure 2:**
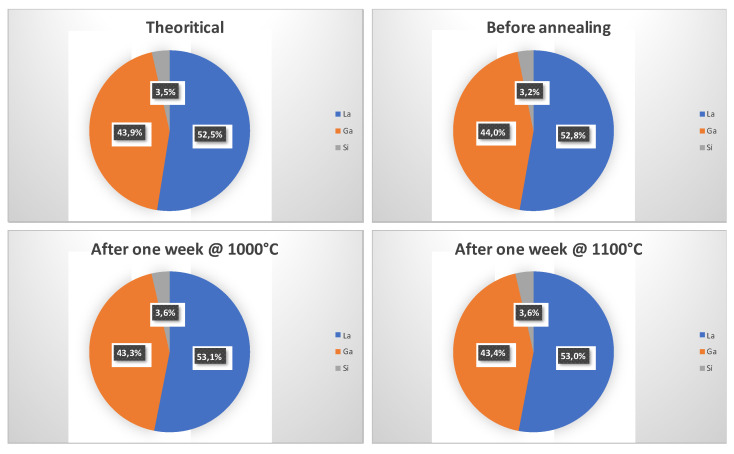
EDXS chemical composition (mass percentage) of reference and annealed LGS crystal surface.

The first important result to mention is that when the annealing time is one week, neither morphological nor chemical modification can be detected at the crystal surface by the SEM and EDXS methods, respectively, as long as the annealing temperature is equal to 1100 °C or less. This result is no longer true when the annealing time is increased to one month, as will be detailed later. The roughness of the sample remains so low that it is not possible to obtain a SEM image with a significant topographic contrast. Only AFM measurements show a very slight evolution of the rms roughness of the surface, which is equal to 3 nm before annealing, reaching 5–7 nm after a one-week annealing process at 1100 °C. This very limited increase in the roughness is likely related to a nanostructuration of the crystal surface occurring during the annealing treatment, consisting of the apparition of regularly spaced nanoripples (Figure 1). Regarding EDXS measurements, the observed variations are not significant since the difference in the mass contents of the La, Ga, and Si elements from one sample to another are lower than the resolution method (Figure 2).

### 3.2. One-Week Annealing Process at 1200 °C

**Figure 3:**
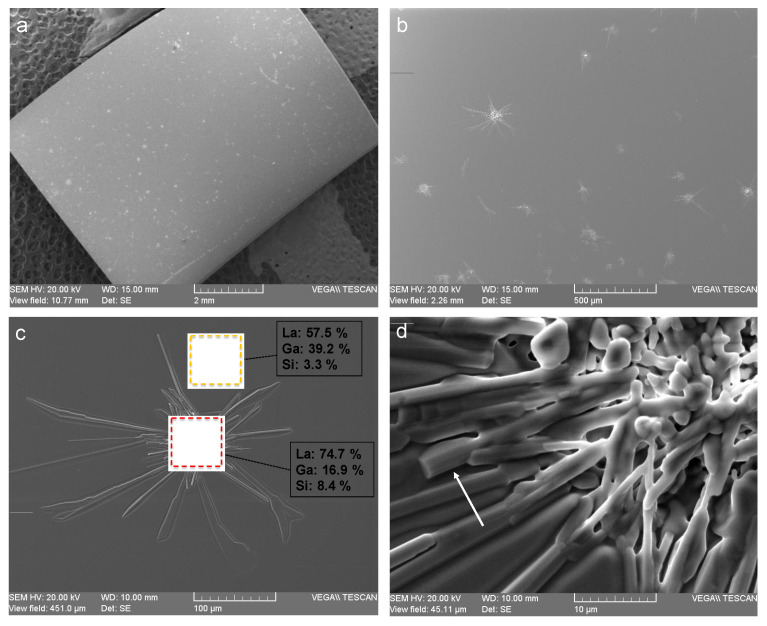
SEM images of the LGS surface after a one-week annealing period at 1200 °C in an air atmosphere. (**a**) Overall image of the substrate: the surface appears sprinkled with isolated defects. (**b**) A closer view of some defects. (**c**) A typical defect. Red and orange squares symbolize the EDXS measurement areas. (**d**) A close-up view of the center of the latter defect. The white arrow indicates a stick with crystalline facets.

The situation becomes totally different if the annealing temperature is increased to 1200 °C (still in the time frame of a one-week process). Many surface defects can be observed on the SEM images (Figure 3a,b). More detailed SEM images of these defects show that they have a corolla-like shape, the typical plane dimensions of which are in the order of 100 µm (Figure 3c). In the vicinity of the defects, the surface chemical composition is slightly altered, and the Ga mass content decreases by some percent, as revealed by the EDXS measurements (Figure 3c). In the center area of the defects, this alteration is much more pronounced, as the Ga mass percentage at the surface is divided by a factor of 2.6 (Figure 3c). A close-up SEM view of this zone reveals a complex morphology, but some sticks with crystalline facets can be observed (Figure 3d).

**Figure 4:**
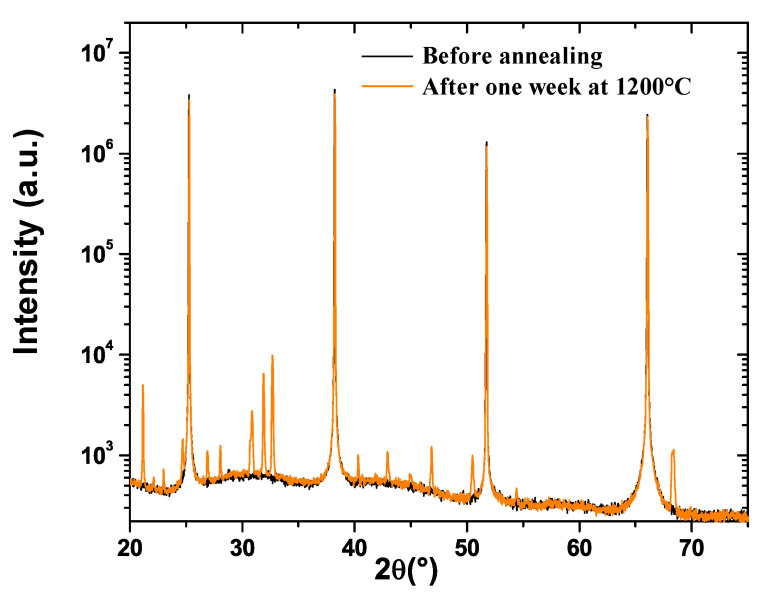
XRD patterns of LGS crystals.

Based on these results, it may be assumed that a new crystalline phase appears within the defects. According to EDXS measurements, this phase would be, similar to LGS, a lanthanum–gallium–silicon oxide compound but with a different stoichiometry, namely a lower proportion of gallium. XRD measurements confirm that a new phase appears during the one-week annealing treatment at 1200 °C (Figure 4). On this diagram, before annealing, only the intense peaks corresponding to the (100) LGS family are visible. After annealing, these peaks are still present and intense, along with numerous new smaller peaks, indicating the presence of one or several new polycrystalline compounds. The positions of almost all of these new peaks are in very good accordance with the theoretical positions of the XRD reflexes of the lanthanum–silicate oxiapatite La_14_Si_9_O_39_ [23] (Table 1). However, this oxiapatite does not contain any gallium atoms, which is a priori not compatible with the EDXS results.

This discrepancy could have two possible origins, possibly occurring at the same time. The first one is that the formed oxiapatite is not exactly La_14_Si_9_O_39_ but a close compound containing a small proportion of gallium. Indeed, Wang and Uda have shown that Ga_2_O_3_ is soluble in small concentrations in La_14_Si_9_O_39_, producing an oxiapatite ternary compound, the chemical formula of which is La_14_Ga_x_Si_9−x_O_39−x/2_ [24]. This assumption is strengthened by the fact that Wang and Uda observed that in such a case, the XRD peaks shift slightly smaller angles when the Ga_2_O_3_ concentration is increased, while within the present study, almost all of the new small XRD peaks are also placed at somewhat smaller angles compared to the corresponding peaks of La_14_Si_9_O_39_ (Table 1).

**Figure 5:**
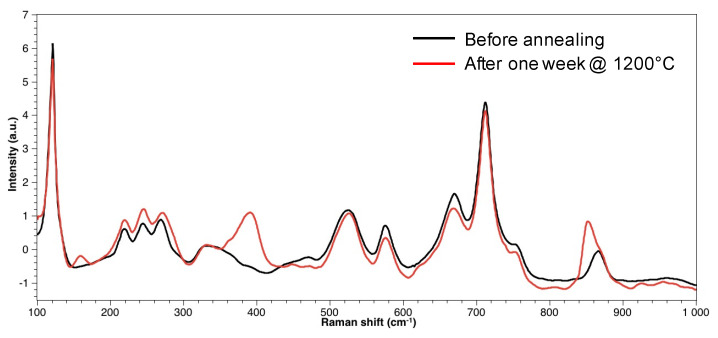
Raman spectra of LGS crystals surface.

**Figure 6:**
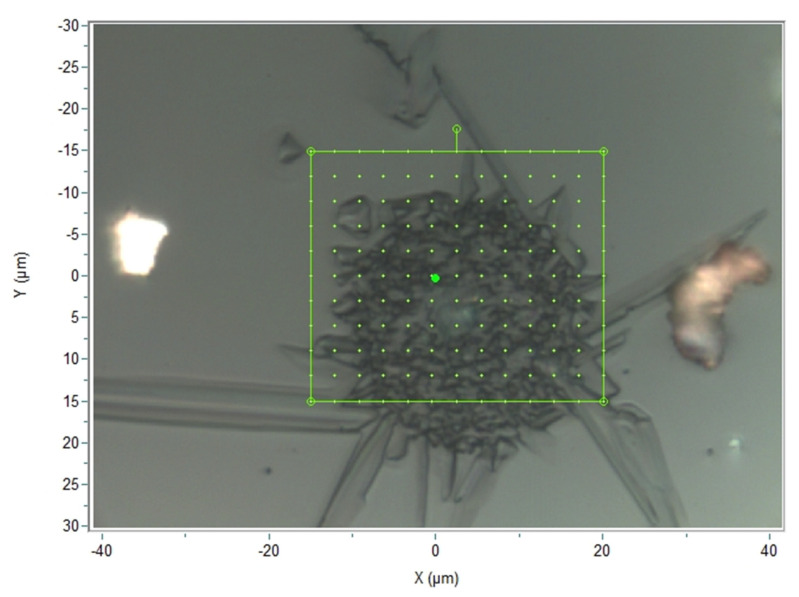
Measurement points for the 3D Raman cartography.

The second possible cause for the detection of Ga in the defects is that their depth is lower than the probed depth by EDXS (~1 µm), so some underlying LGS is also measured by EDXS. In order to assess this hypothesis, Raman measurements were performed to determine the in-depth extension of the defects. First of all, the Raman spectra obtained from a flawless LGS crystal surface and those obtained from a defect zone were compared (Figure 5). It appears that both spectra are quite similar, but the spectrum related to the defects shows three specific peaks located at 160, 390, and 850 cm^−1^. Based on this result, a 3D Raman cartography of a typical defect area was realized: to do so, Raman spectra were first measured at 99 chosen points of the substrate surface (Figure 6). Then, the process was repeated, focusing the excitation laser at various successive depths: namely 1, 2, 3, 4, and 6 µm. Consequently, 594 Raman spectra were obtained in total. For each spectrum, the intensity for a Raman shift of 850 cm^−1^ was measured. Then, color coding was defined in order to map this intensity, which was directly related to the presence of the oxiapatite phase.

**Figure 7:**
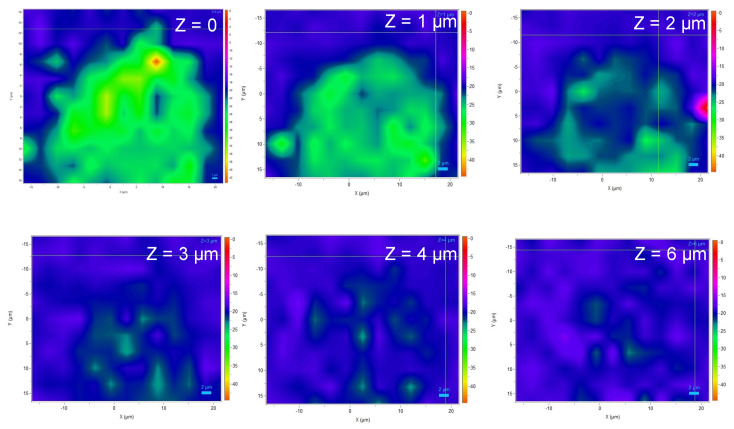
Raman cartography of a typical defect (each map refers to a particular depth, Z).

The obtained maps suggest that the defects have an in-depth extension that is likely larger than 1 µm and lower than 2 µm (Figure 7). Indeed, the spatial resolution of the method, due to the wavelength of the excitation laser (532 nm), is around 1 µm. Thus, the poor traces of oxiapatite visible on the map for a depth of 2 µm (Figure 7) are likely a marker of the presence of La_14_Ga_x_Si_9−x_O_39−x/2_ at a depth around 1.5 µm. In conclusion, the in-depth extension of the defects seems a bit larger than the probed depth conducted by EDXS (~1 µm), so it is likely that the defects are made of an oxiapatite ternary compound, the chemical formula of which is La_14_GaxSi_9−x_O_39−x/2_. EDXS measurements conducted at the submicronic level (using transmission electron microscopy) would be necessary to accurately determine the value of the x factor.

### 3.3. One-Month Annealing Process at 1000 °C

**Figure 8:**
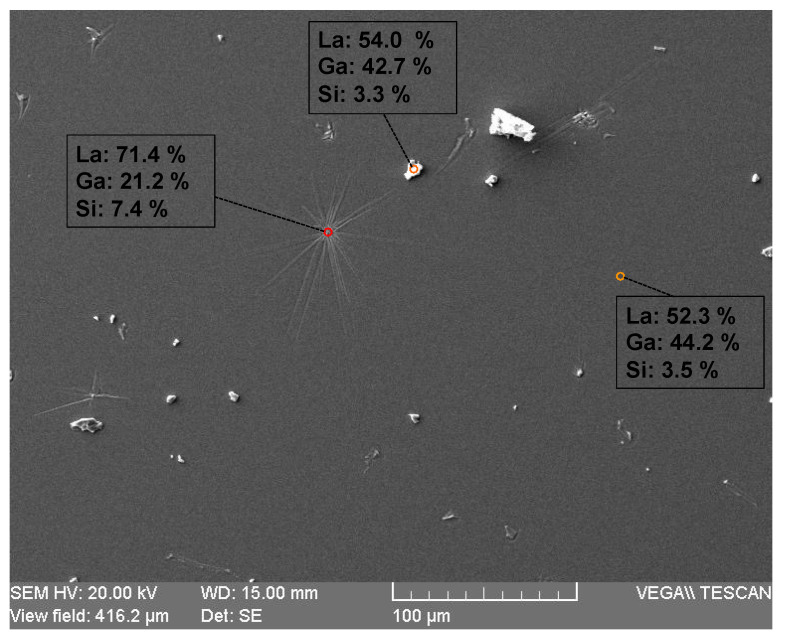
SEM image of LGS surface after a one-month annealing period at 1000 °C in an air atmosphere. Colored spots indicate the locations of EDXS measurements.

Based on the entirety of the former results, it can be stated that LGS surface is stable at high temperatures in an air atmosphere for one week, provided that the temperature does not exceed 1100 °C. In order to study the limitations of LGS for longer applications, some samples were annealed for one month at 1000 °C in air atmosphere. After this treatment, the LGS surface presented many flaws (as a reminder, this was not the case after one week at this temperature). However, the SEM images reveal three main differences in comparison to the surface state after one-week of annealing at 1200 °C. The first one is that the density of the flaws is much larger after one month of annealing at 1000 °C. However, at the same time, these flaws appear to be much smaller (Figure 3c and Figure 8). Moreover, many out-of-plane defects are visible, which was not the case at 1200 °C. EDXS measurements show that the in-plane defects have a composition close to that of the defects observed at 1200 °C, with a Ga mass percentage at the surface that is roughly twice as small as it is in LGS (Figure 8). Regarding the out-of-plane defects, their composition is really very close to that of LGS. It may be assumed that these flaws could result from a recrystallisation process occurring during the annealing treatment.

In order to investigate the consequences of this deterioration on the surface acoustic properties of LGS, SAW measurements were realized. It was decided that this experiment would not be conducted at high temperature because the results would have been significantly influenced by the degradation of the electrodes, which would be severe during a one-month annealing process at 1000 °C, regardless of the nature of these electrodes [25,26]. Consequently, identical SAW delay lines were realized on the substrates annealed for one month at 1000 °C and, for comparison, on non-annealed substrates. Both kind of devices were then measured at room temperature. In both cases, careful attention was paid so that the electrodes were strictly identical in terms of thickness and orientation in order to avoid any influence of these parameters on the results.

**Figure 9:**
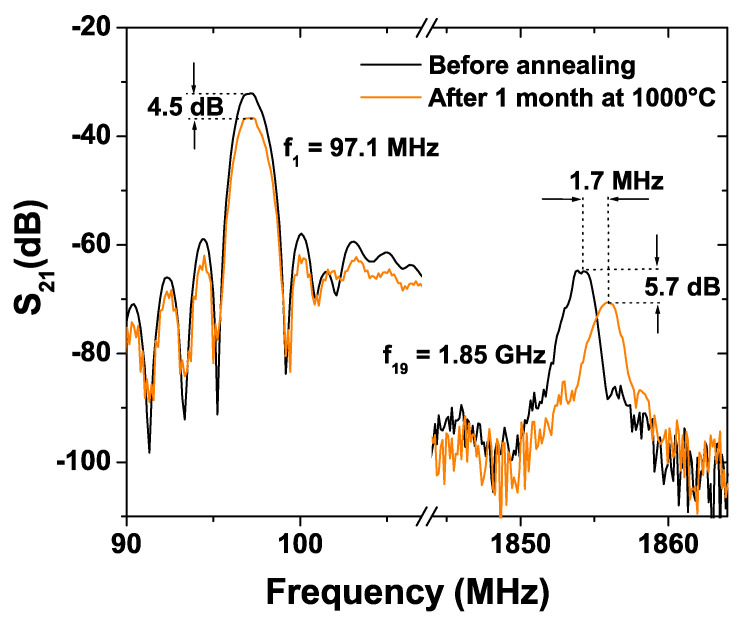
S_21_ magnitude frequency response (1st and 19th harmonics) of SAW delay lines built from reference and annealed LGS crystals.

The design of the delay lines was chosen so that numerous harmonics could be generated in order to study the influence of the wavelength, and thus the wave penetration depth, on the acoustic properties of the annealed substrate. In practice, it was possible to generate SAW from the fundamental at 97 MHz (λ = 28 µm) up to the 19th harmonic centered at 1.854 GHz (λ = 1.47 µm). Two kinds of effects related to the annealing process undergone by the substrate were observed. Both the insertion losses and the operating frequencies are larger in the case of the annealed substrates (Figure 9). In the case of the insertion losses, the phenomenon seems independent of the frequency and relatively moderate, the difference generally being some dBs (Table 2). On the contrary, the shift in the frequency increases with the harmonic rank. of the harmonic rank is only 0.13 MHz in the case of the fundamental mode (at 97 MHz), whereas it reaches 1.7 MHz for the 19th harmonic (Figure 9). This effect could have two origins a priori. It could either simply be related to the decrease of the wavelength with the harmonic rank, or to the apparition of the above-described defects at the surface of the LGS which would modify the velocity of the SAW, especially since the penetration depth of the waves decreases.

Thus, the SAW velocity was calculated for each harmonic (Table 2). Values in the range of 2720–2735 m/s were found, which is in good accordance with the literature for YX orientation [27]. It appears that for both kinds of substrates (annealed and non-annealed), the SAW velocity slightly increases with the harmonic rank, which is related to the mass loading effect of the aluminium electrodes (in that case, the mass loading effect leads to an increase of the velocity when the relative thickness of the electrodes rises since the SAW velocity in aluminium is larger than in YX LGS). Moreover, it can be observed that the SAW velocity is only slightly larger, by about 1–2 m/s, in the case of the annealed substrates in comparison with non-annealed substrates (Table 2), independently of the harmonic rank. This confirms (together with the results related to the insertion losses) that the acoustic properties of the LGS surface are modified during the one month annealing process at 1000 °C.

However, these modifications seem constant at a deepness range of some hundreds of nanometers to some tens of micrometers. Consequently, this phenomenon cannot be attributed to the apparition of defects at the surface of the crystal since their typical deepness is likely less than one micrometer for the one-month annealing process. It is more likely that the density or the elastic constants of the LGS crystal are modified by the annealing process at 1000 °C on a deepness that is still to determine but that is larger than some tens of micrometers. Finally, it has to be noted that that such a difference in SAW velocity, a difference of of 1–2 m/s, would induce a frequency shift in the 434 MHz ISM band (corresponding to a wavelength around 6.3 µm) of approximately 0.2 MHz, that is to say 450 ppm. Since the temperature coefficient of the frequency of YX LGS is around −45 ppm/°C at 1000 °C [6], these modifications of the acoustic properties of the LGS surface could induce temperature measurement errors close to 10 °C, which is quite significant.

## 4. Conclusions

The investigations conducted in this study show that the LGS surface is stable under a high-temperature air atmosphere, depending on both the temperature and the process duration. If the annealing process is one week long, the LGS surface is stable up to 1100 °C. Surface defects appear at 1200 °C. Their typical in-plane size after one week is 100 µm. XRD and Raman spectroscopy suggest that the deepness of these defects is likely larger than 1 µm and lower than 2 µm and that they are constituted by an oxiapatite ternary compound, the chemical formula of which is La_14_Ga_x_Si_9−x_O_39−x/2_. If the annealing process is longer, namely one-month long, surface defects are visible on the substrates heated at 1000 °C. SAW investigations show that the one-month annealing process at 1000 °C slightly modifies the acoustic properties of the LGS surface at a deepness of some tens of micrometers at least. This phenomenon is likely not related to the apparition of the defects at the crystal surface since they are much less deep, but they more than surely create to a slight change to the LGS density, elastic, and piezoelectric constants close to the surface. It was finally evaluated that in the case of LGS-based SAW sensors operating in the 434 MHz ISM band, these modifications to the LGS surface acoustic properties can cause temperature measurement errors of around 10 °C. 

Ongoing experiments should help to achieve a better understanding of these deterioration phenomena and to provide answers to the many associated remaining questions, such as those about the origins of the formation of the defects (nucleation process, importance of the surface orientation, etc.) or the kinetics of their growth.

## Figures and Tables

**Table 1:** Experimental and theoretical (La_14_Si_9_O_39_) diffraction angles.

Experimental reflexes positions (°)	21.15	22.12	23.00	24.71	26.90	28.06	30.88	31.89
Theoretical La_14_Si_9_O_39_ reflexes positions (°)	21.12	22.08	-	24.77	26.96	28.05	30.93	31.89
La_14_Si_9_O_39_ plans family	(200)	(111)	-	(002)	(102)	(210)	(112)	(300)
Experimental reflexes positions (°)	32.69	40.31	42.92	44.90	46.85	50.49	54.41	68.34
Theoretical La_14_Si_9_O_39_ reflexes positions (°)	32.77	40.61	42.97	45.02	47.05	50.77	-	68.68
La_14_Si_9_O_39_ plans family	(202)	(311)	(400)	(222)	(320)	(004)	-	(404)

**Table 2:** Main results from SAW measurements.

	Harmonic Rank	1	3	7	9	13	15	19
Non-annealed substrates	Frequency (MHz)	97.15	291.90	681.58	876.16	1267.2	1461.8	1854.2
	Insertion losses (dB)	−32.1	−37.4	−41.5	−48.5	−49.6	−58.3	−64.9
	V_SAW_ (m/s)	2720	2724	2726	2726	2729	2729	2733
Annealed substrates (1 month @ 1000 °C)	Frequency (MHz)	97.28	291.965	681.66	876.54	1268.0	1463.1	1855.9
	Insertion losses (dB)	−36.6	−42.5	−44.2	−55.6	−53.5	−69.2	−70.6
	V_SAW_ (m/s)	2724	2725	2727	2727	2731	2731	2735
